# Erenumab for Migraine Prevention in a 1-Year Compassionate Use Program: Efficacy, Tolerability, and Differences Between Clinical Phenotypes

**DOI:** 10.3389/fneur.2021.805334

**Published:** 2021-12-10

**Authors:** Jean Schoenen, Gregory Timmermans, Romain Nonis, Maïté Manise, Arnaud Fumal, Pascale Gérard

**Affiliations:** Headache Research Unit, Department of Neurology, Citadelle Hospital-Liège, University of Liège, Liège, Belgium

**Keywords:** migraine prophylaxis, monoclonal antibodies blocking CGRP transmission, erenumab, compassionate use, outcome predictors

## Abstract

During a 1-year compassionate use program, 156 patients with migraine self-administered a monthly dose of erenumab 140 mg with a subcutaneous autoinjector. Main inclusion criteria were: ≥ 4 migraine days/month and ≥two prior prophylactic treatment failures. The patients covered the migraine severity spectrum from episodic migraine (EM) (*n* = 80) to chronic migraine (CM) (*n* = 76). During the 3rd month of treatment, monthly headache days decreased by 45.7% in EM and 35.5% in CM. The 50% responder rate for reduction in monthly headache days was significantly higher in EM (55%) than in CM (43%) (*p* = 0.05). In both the migraine subgroups, the clinical improvement vs. baseline was already significant during the 1st month of treatment (*p* < 0.001). There were also significant reductions in mean headache severity, duration, and monthly days with acute drug intake. The 30% responder rate at 3 months was 60% in CM and 54.1% of patients reversed from CM to EM. The therapeutic effect was maintained at 12 months when 50% responder rates, considering discontinuation for lack of efficacy or adverse effects as 0% response, still were 51% in EM and 41% in CM. A total of 10 patients with EM (12.5%) and 23 patients with CM (30.3%) had discontinued treatment, considering the treatment as ineffective. At 3 months, 48% of patients reported non-serious adverse events among which the most frequent was constipation (20.5%); corresponding figures at 12 months were 30 and 15%. Discontinuation due to an adverse effect for the entire 12 month period was rare (3.8%). The lower efficacy in CM than in EM was mainly due to a very low 50% responder rate in patients with CM with continuous pain (13%) as compared to CM with pain-free periods (58%) (*p* < 0.001). Similarly, the 50% responder rate was lower in patients with ≥two prior prophylactic treatment failures (40.5%) compared to those with two failures (70%) (*p* < 0.05). There was no significant efficacy difference between low (4–7 migraine days/month, *n* = 22) and high frequency (8–14 days, *n* = 59) EM nor between patients with CM with (*n* = 50) or without (*n* = 26) acute medication overuse. Erenumab had no effect on the frequency of auras. Taken together, erenumab 140 mg monthly was highly effective for migraine prophylaxis over the whole severity spectrum of the disease, except in patients with continuous headaches. Its effect is significant after the first injection, quasi-maximal after the second injection, and does not wear off after 12 months. The most frequent adverse effect was constipation. These results are compared to those published for erenumab in the pivotal randomized placebo-controlled trials and to those reported in several recent real-world studies.

## Introduction

The monoclonal antibodies (mAbs) blocking calcitonin gene-related peptide (CGRP) or its receptor (CGRP/rec) are a major breakthrough in the prophylactic treatment of migraine. They are the successful translation into clinical practice of more than 35-years of basic and clinical research in migraine (1). Since 2014, randomized controlled trials (RCTs) of the four hitherto available molecules (eptinezumab, erenumab, fremanezumab, and galcanezumab) have collectively included several thousands of patients, making them the most extensively studied class of prophylactic migraine treatments. Their results (2) and those of subsequent *post-hoc* studies analyzing effect size, effect onset, sustainability, response in subgroups of difficult-to-treat patients, and long-term safety and tolerability (3) have been reviewed previously.

Despite a large placebo response in all the trials (4), the trial results demonstrate that the CGRP/rec mAbs are clearly superior to placebo with average placebo-subtracted 50% responder rates for reduction in monthly migraine headaches of 21.4% in episodic migraine (EM) (number needed to treat: 4–5) and 17.4% in chronic migraine (CM) (number needed to treat: 4–8). The effect starts within 1 week and is sustained for long periods, up to 5-years for erenumab (5); for erenumab and galcanezumab, it outlasts treatment termination for no more than 12 weeks (6). According to several trials or subanalyses (3), CGRP/rec mAbs, including erenumab, are effective even in “difficult-to-treat” patients with prior failure of several prophylactic treatments and/or with medication overuse. They significantly reduce disability (7) and healthcare resource utilization (8). In the pivotal placebo-controlled RCTs, the incidence of adverse effects with CGRP/rec mAbs is close to that of placebo, except the possible occurrence of injection site reactions.

Despite the lack of direct comparative trials with available prophylactic treatments, the added value of CGRP/rec mAbs for reduction of the individual and societal burden of migraine was questioned in health technology assessments (NICE 2021 for erenumab) (9) and cost-effectiveness studies (10). Considering published RCTs of migraine prophylactics, it was argued that, compared to the most effective classical treatments such as topiramate, the CGRP/rec mAbs stand out more by their unprecedented efficacy over tolerability profile (3) and a much greater likelihood to help than to harm (11) than by superior efficacy.

Erenumab, the CGRP/rec mAb blocking the CGRP receptor, was approved by the US Food and Drug Administration (FDA) on May 17, 2018 and by the European Medicines Agency (EMA) on July 26, 2018 on the basis of one pivotal RCT “STRIVE” (12) and one supportive RCT “ARISE” (13) in EM and one RCT in CM (14). A dedicated RCT “LIBERTY” was also performed in patients with prior failure of two to four preventive drug treatments (15) (Table 1). Erenumab is commercially available in Belgium only since June 1, 2021. Before commercial availability, Novartis Pharma NV (Vilvoorde, Belgium, UK) decided to setup a compassionate use program in order to make erenumab available to a group of adult migraine patients who are in need of prophylaxis and for whom no acceptable alternative prophylactic treatment is available. The program was approved by the Belgian Agency for Medicines and Health Products on November 11, 2018 and accessible to Belgian neurologists under their full responsibility. We felt that this program offered a unique opportunity to provide evidence informing patients and their treating physicians on how erenumab would perform with respect to tolerability, safety, and efficacy outside of the strict confines of a RCT, i.e., in a quasi-real-world scenario. In this study, we will present the results observed in our tertiary headache center and focus on tolerability and clinical variables that may influence treatment outcome.

**Table 1:** Pivotal randomized placebo-controlled trials of erenumab in migraine.

**Trial patients**	**Treatment modalities**	**Endpoints**	**Outcome (erenumab vs. placebo)**
**Episodic migraine**			
Dodick et al. (ARISE) (13) 577 adults 8 monthly migraine days on average	70 mg subcut monthly vs. placebo	Primary: change in monthly migraine days (MMD) during DB phase weeks 9–12 vs. baseline	−2.9 vs. −1.8 MMD Mean difference (95% CI): −1.0 (−1.6/−0.5)
Duration of double-blind (DB) phase: 12 weeks		Secondary : ≥50% responder rate	39.7% vs. 29.5% Odds ratio (95% CI) 1.59 (1.12/2.27)
Goadsby et al. (STRIVE) (12) 955 adults 8 monthly migraine days on average Duration of DB phase: 24 weeks	70 mg subcut monthly vs. placebo 140 mg subcut monthly vs. placebo	Primary: change in monthly migraine days (MMD) during DB phase weeks 13–24 vs. baseline Secondary: ≥50% responder rate Primary: change in monthly migraine days (MMD) during DB phase weeks 13–24 vs. baseline Secondary: ≥50% responder rate	−3.2 vs. −1.8 MMD Mean difference (95% CI): −1.4 (−1.9/−0.9) 43.3% vs. 26.6% Odds ratio (95% CI) 2.13 (1.52/2.98) −3.7 vs. −1.8 MMD Mean difference (95% CI): −1.9 (−2.3/−1.4) 50% vs. 26.6% Odds ratio (95% CI) 2.81 (2.01/3.94)
**Chronic migraine**			
Tepper et al. (14) 667 adults 18 monthly migraine days on average Duration of DB phase: 12 weeks	70 mg subcut monthly vs. placebo 140 mg subcut monthly vs. placebo	Primary: change in monthly migraine days (MMD) during DB phase weeks 9–12 vs. baseline Secondary: ≥50% responder rate Primary: change in monthly migraine days (MMD) during DB phase weeks 9–12 vs. baseline Secondary: ≥50% responder rate	−6.6 vs. −4.2 MMD Mean difference (95% CI): −2.5 (−3.5/−1.4) 40% vs. 23% Odds ratio (95% CI) 2.2 (1.5/3.3) −6.6 vs. −4.2 MMD Mean difference (95% CI): −2.5 (−3.5/−1.4) 41% vs. 23% Odds ratio (95% CI) 2.3 (1.6/3.5)
**Episodic or chronic migraine with preventive treatment failures**			
Reuter et al. (15) 226 adults 9 monthly migraine days on average Duration of DB phase: 12 weeks	140 mg subcut monthly vs. placebo	Primary: ≥50% responder rate during DB phase weeks 9–12 vs. baseline Secondary: change in monthly migraine days (MMD) during DB phase weeks 9–12 vs. baseline	30% vs. 14% Odds ratio (95% CI) 2.7 (−1.4/−5.2) −1.8 vs. −0.2 MMD Mean difference (95% CI): −1.6 (−2.7/−0.5)

## Subjects and Methods

### Eligibility Criteria of the Patients

The following criteria had to be fulfilled for the provision of the compassionate use program: (1) an independent request from the treating neurologist; (2) patients with a serious condition and no comparable or satisfactory alternative approved nor commercially available therapy in Belgium; (3) patients not eligible for a clinical trial running with erenumab; (4) a potential patient benefit to justify the potential risk of treatment use; and (5) access provision allowed as per local laws and regulations.

Patients eligible for inclusion had to meet all the following criteria (Table 2): age 18–65-years old; history of episodic or CM with or without aura for 12 months (International Classification of Headache Disorders - ICHD-3 1.1, 1.2.1, or 1.3 criteria) (16); **≥**4 migraine days per month; a documented history of prior failure of ≥ two prophylactic drug treatments, among which at least one beta-blocker, unless contraindicated (propranolol, metoprolol, atenolol, bisoprolol, timolol, topiramate, valproate, amitriptyline, venlafaxine, flunarizine, or candesartan); no comparable or satisfactory alternative therapy available for migraine prophylaxis; no history of frequent tension-type headaches; no history of hypersensitivity to any drug of similar chemical classes as erenumab; no myocardial infarction, stroke, transient ischemic attack, and unstable angina within 12 months prior to treatment; no coronary artery bypass surgery or other revascularization procedures within 12 months prior to treatment; no history or current diagnosis of ECG abnormalities indicating significant safety risk as assessed by the treating physician; no participation in a prior investigational study within 30 days prior to enrollment or within five half-lives of the investigational product, whichever was longer; for female patients: no pregnancy or lactation and reliable contraception, if needed. All the included patients were naïve to treatment with CGRP/rec mAbs.

**Table 2:** Characteristics and eligibility criteria of the patients.

**TOTAL NUMBER OF PATIENTS** (n = 156) 02/2019-12/2019
Erenumab 140 mg subcut/month
Total migraine: *n* = 156 (mean age: 44 ± 12 y; 128 F, 28 M)
Episodic migraine (ICHD-3 1.1): *n* = 80 (mean age: 45 ± 10 y; 65 F, 15 M)
Low frequency (<8 d/month): *n* =
Chronic migraine (ICHD-3 1.3): *n* = 76 (mean age: 44 ± 13 y; 63 F, 13 M)
Pain-free periods (ICHD-3 A1.3.1): *n* = 46
Continuous pain (ICHD-3 A1.3.2): *n* = 30
Migraine with aura (ICHD-3 1.2.1): *n* = 37 (*n* = 19 EM; *n* = 18 CM)
Disease duration: 18 ± 6 y
Duration of chronic migraine: 5 ± 3 y
2 prior preventive treatment failures: *n* = 40
> 2 prior preventive treatment failures: *n* = 116
**INCLUSION CRITERIA**
• Age: 18–65-years • Migraine without or/and with aura (ICHD-3 1.1, 1.2.1, 1.3) • ≥4 migraine days/month • ≥2 preventive treatment failures (among which a beta-blocker) • no contraindication, no pregnancy

A written informed consent was obtained from all the patients prior to the start of the treatment and the program was approved by the local Ethics Committee.

### Visits and Clinical Assessments

At the first visit, eligible patients were invited to benefit from the compassionate use of erenumab and received oral and written information about the compound and the protocol of the program. The physician declaration, treating physician attestation, and managed access program information forms were then forwarded to the provider who, in case of acceptance, sent the drug to the hospital pharmacy within 10 days for storage at 2–8°C. During the second visit, 8–15 days later, the patients gave their written informed consent and were instructed how to use the erenumab (Aimovig) autoinjector. They received the first subcutaneous injection of 140 mg in the outpatient clinic where they were asked to wait for 15 min before returning home to rule out a possible systemic allergic reaction. Two (at the second visit) or three Aimovig autoinjectors (at each subsequent visit) were given in an isothermal bag with a cold pack to the patients, who were instructed to store them at 2–8°C and to inject 140 mg of the drug at room temperature every 28 (±2) days.

For 1-year, followed-up visits were scheduled every 3 months for a clinical examination, i.e., with blood pressure measurements, weight control, collection of headache diaries, interview about occurrence of adverse effects, and delivery of three 140 mg autoinjectors for the next trimester. Patients could call the headache clinic emergency number at any time, if there was an unexpected event during the program.

Before inclusion in the program, all the patients had filled in a paper headache diary for ≥1 month and they had to pursue this for the whole duration of the program. The following parameters were monitored in the diaries: headache occurrence; intensity (on a 1-mild to 3-severe scale); duration; presence of nausea or vomiting; sensitivity to light, noise or odors; intake and number of acute migraine drugs; and for females with ovarian cycles, days of menstruation. At the end of each trimester, patients were asked if they were satisfied with the treatment and if they would recommend it to others.

### Statistics

GraphPad PRogramming In Statistical Modeling (PRISM) version 8.01 software.

The following variables were expressed as mean ± SEM and analyzed: monthly number of headache days (MHD), proportion of patients having ≥50% reduction in MHD, headache severity and duration per headache day, monthly number of days with intake of acute medication, and total number of acute medications taken per month. Missing diary data were rare; if they occurred, they handled as the last value carried forward.

We chose not to analyze headache and migraine days separately for the following reasons. In patients with EM, the majority of headaches not fulfilling all the ICHD migraine criteria seem to have a migrainous pathophysiology according to the Spectrum study (17). In CM, headaches not fulfilling all the migraine criteria are frequent and contribute to disability (16). Moreover, during effective prophylactic treatments, persistent attacks may have reduced severity and loose associated migraine-specific features.

The normality of all the variables was verified with the Shapiro–Wilk test. Since no variable had a normal distribution, we chose the Friedman's ANOVA with the *post-hoc* Dunn's test as non-parametric statistical method. The chi-squared test was used to compare subgroups of the patients.

Significance level was set at *p* < 0.05.

## Results

### Disposition and Clinical Characteristics of the Patients

Disposition and clinical characteristics of the patients are given in Table 2. Between February 2019 and December 2019, 156 patients consulting our tertiary headache clinic at Liège University (Citadelle Hospital, Liège, Belgium UK) were included in the program: mean age 44 ± 12-years; 128 females, 28 males.

A total of 80 patients suffered from EM without aura (ICHD-3 1.1) and 76 patients suffered from CM (ICHD-3 1.3). A total of 37 patients in both the groups combined (24%) also had attacks of migraine with aura (ICHD-3 1.2.1): 19 EM and 18 CM.

The majority (74%) of patients with EM had high frequency migraine, i.e., 8–14 days/month.

Among patients with CM, 46 (61%) patients had ≤ 25 headache days/month and pain-free periods (ICHD-3 A1.3.1), while 30 (39%) patients had continuous pain (ICHD-3 A1.3.2) (16). A total of 50 out of 76 patients (66%) with CM had ongoing acute medication overuse according to the ICHD-3 criteria 8.2.2, 8.2.3, or 8.2.5 (16).

As expected from the inclusion criteria, the cohort of patients enrolled in the program mainly covered the two most disabling tiers of severity spectrum of migraine: high-frequency EM (HFEM) and high-frequency CM.

Within the total cohort of patients and as per eligibility criteria of the patients, 40 (26%) patients had failed on two prior prophylactic drugs, whereas the majority (*n* = 116, 74%) had prior experience of more than two treatment failures. None of them had received onabotulinumtoxin A.

Most patients (68%) had interrupted prophylactic treatment at least 3 months before participating in the program. A total of 66 (42%) patients were taking a prophylactic migraine drug at the time of entering the program, 46 patients in the CM group, and 20 patients in the EM group. They were asked to continue their prophylaxis at the same dosage during the 1st trimester following program entry, after which a progressive withdrawal could be attempted depending on the clinical evolution.

### Outcome After 3 Months

We will first describe in detail the outcome after 3 months of treatment, as retention of patients was 100% during this period and most RCTs of erenumab have lasted 3 months.

**Figure 1:**
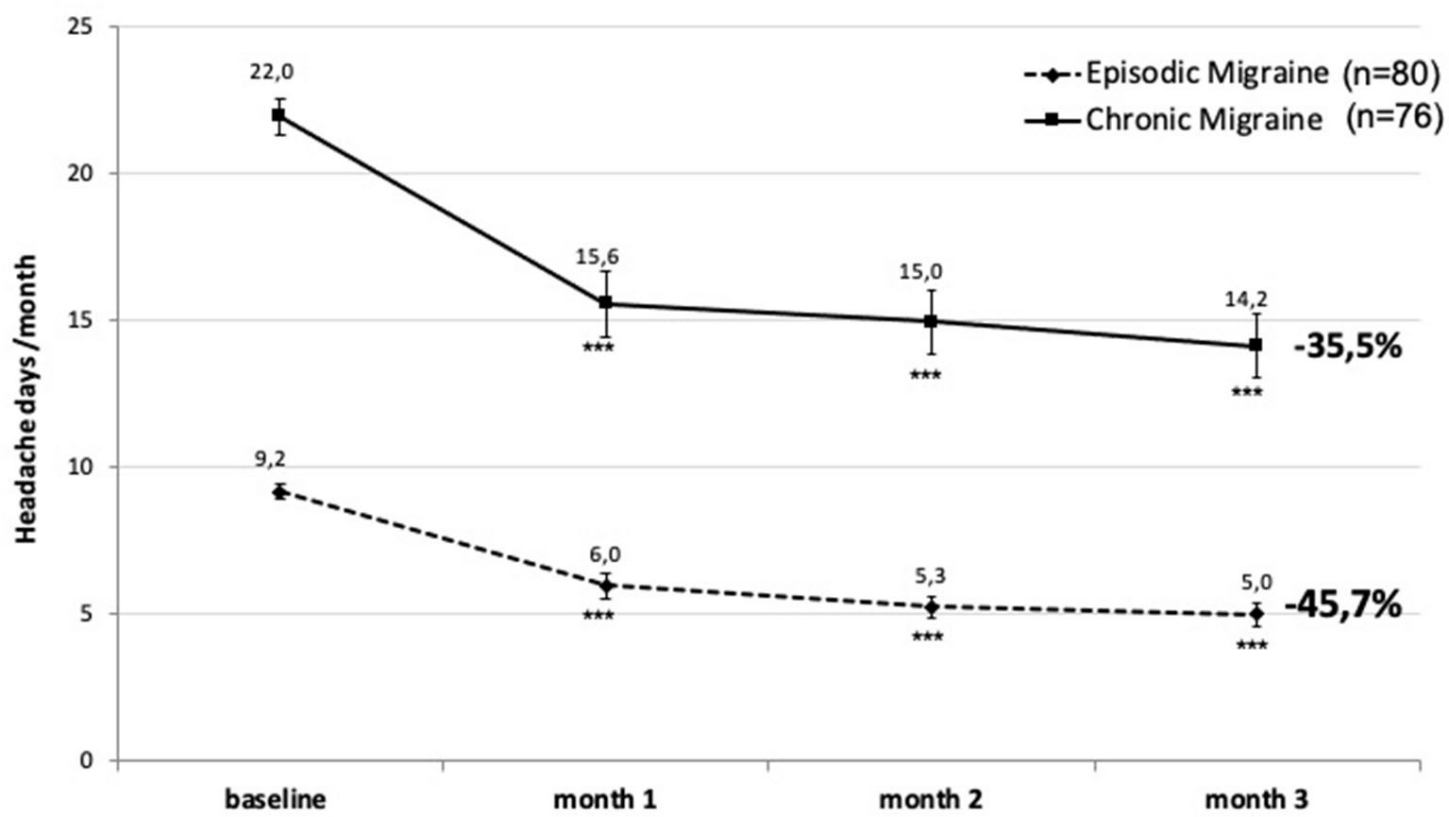
Change in monthly headache days after 3 months of erenumab treatment in patients with episodic migraine (EM) (dashed line) and chronic migraine (CM) (continuous line). ***p < 0.001 vs. baseline (Friedman's ANOVA, the *post-hoc* Dunn's test).

During the 3rd month after monthly injections of 140 mg erenumab, MHD decreased from a mean of 9.2 before treatment to 5 (−4.2 MHD; −45.7%) (*p* < 0.001) in patients with EM and from 22 to 14.2 (−7.8; −35.5%) (*p* < 0.001) in CM. The reduction was significant as soon as 1 month after the first injection (Figure 1).

**Figure 2:**
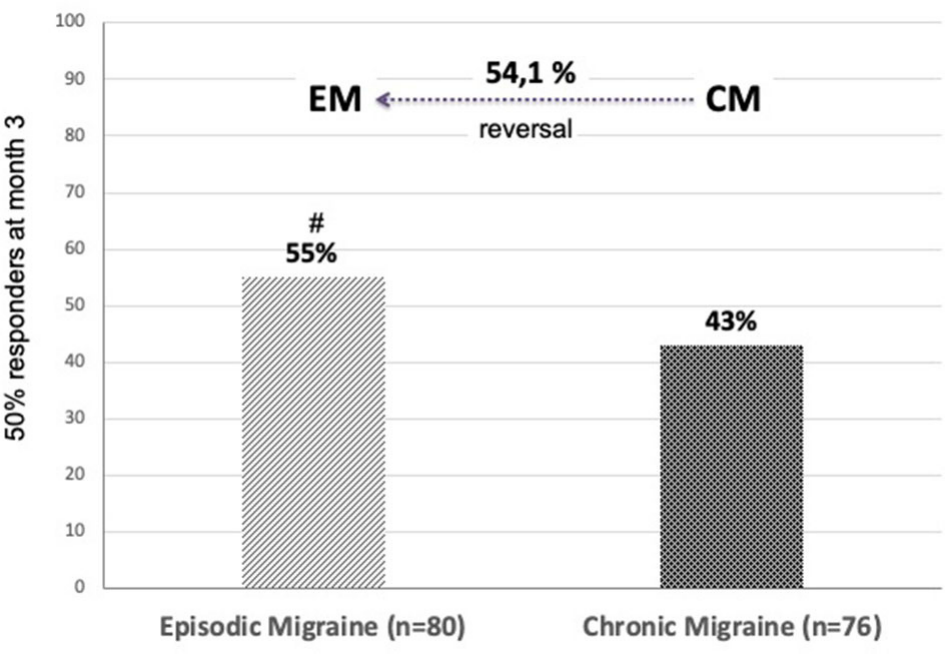
Proportion of patients with ≥50% reduction in monthly headache days during 3 months of treatment with 140 mg erenumab per month. At 3 months, 54.1% of patients with CM had reverted to EM. #*p* = 0.05 chi-squared test “EM vs. CM”.

The proportion of patients having at least a 50% decrease of MHD during 3 months compared to the pretreatment month (baseline) was 55% in EM and 43% in CM and a significant difference (*p* < 0.05). At the end of 3 months, more than half of patients with CM had reverted to an EM pattern (Figure 2).

**Figure 3:**
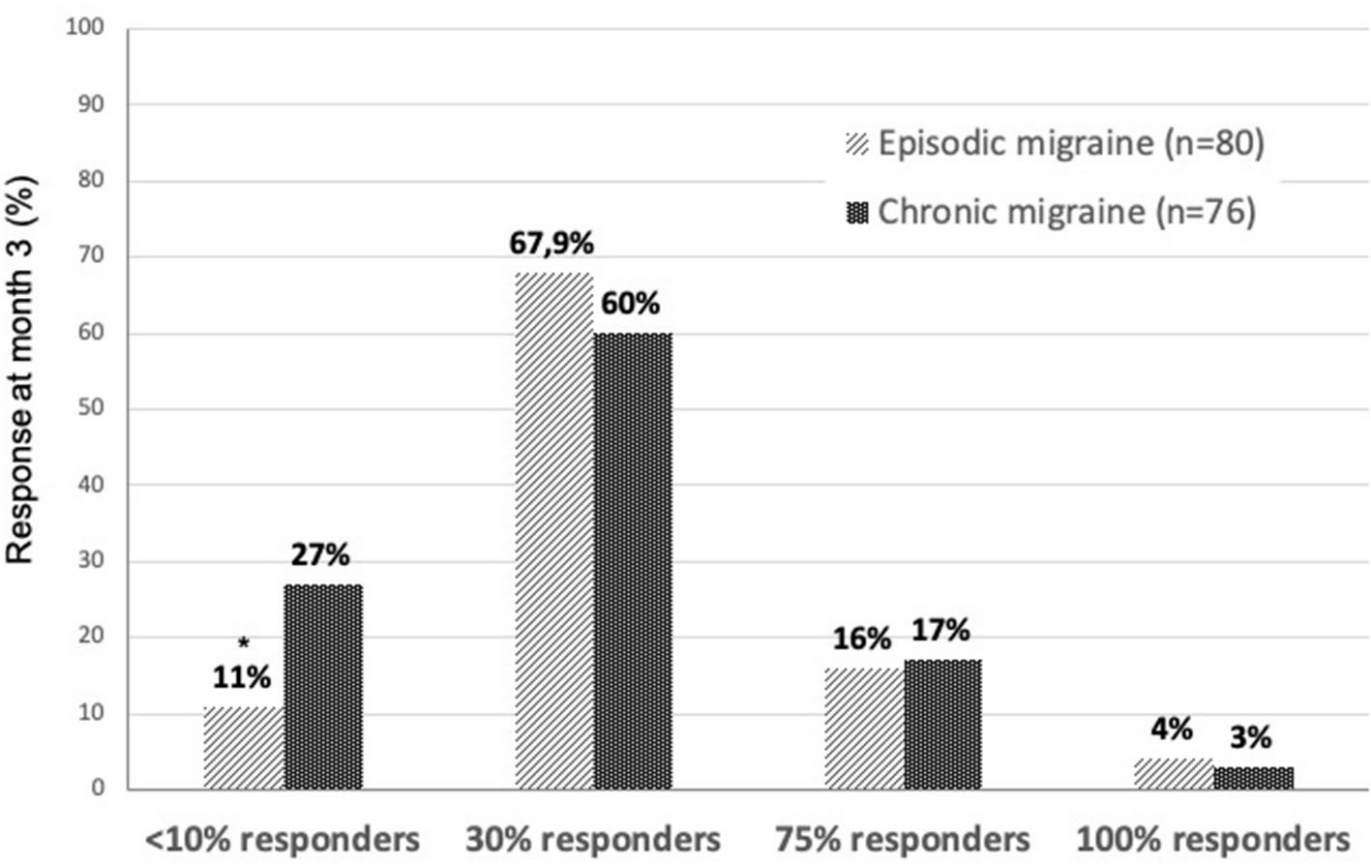
Proportions of different levels of decrease in monthly headache days during 3 months of erenumab treatment in EM (light bars) and CM (dark bars). *p = 0.014 chi-squared test “EM vs. CM”.

The 30% responder rate, a less stringent outcome measure often used in difficult-to-treat patients with chronic headache, was 60% in CM, not significantly different from the 67.9% found in EM. Non-responders having <10% decrease in MHD were more numerous in the CM (27%) than in the EM group (11%) (*p* = 0.014). Few patients (4 and 3% of patients with EM and CM, respectively) had total disappearance of headaches in the 3rd month of treatment (Figure 3).

**Figure 4:**
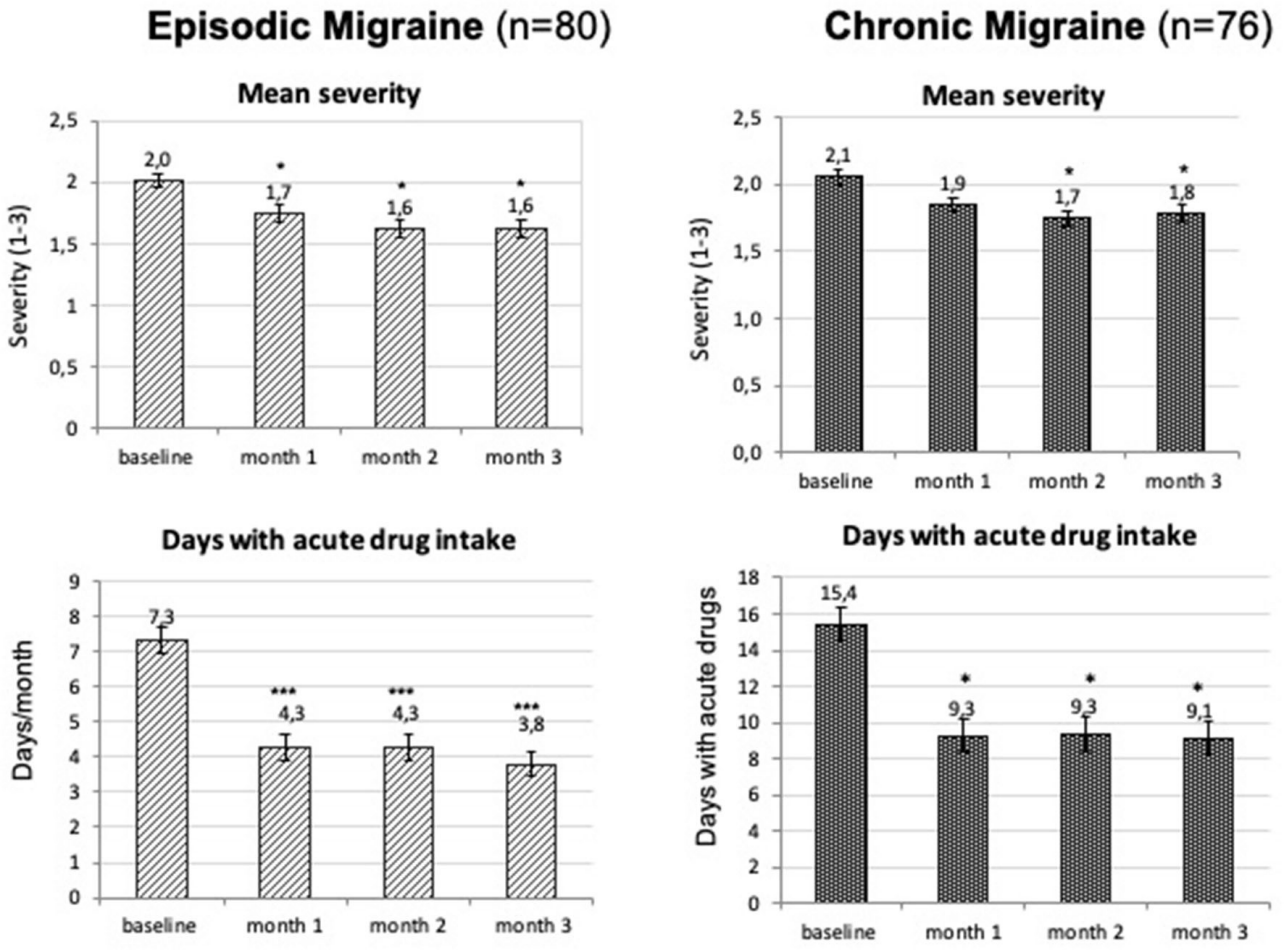
Mean monthly headache severity (three-point scale from 1-mild to 3-severe headache) and number of days with acute drug intake (mean ± SEM) during the first 3 months of treatment. *p < 0.05; ***p < 0.001 (Friedman's ANOVA, the Dunn's *post-hoc* test).

Other headache features also improved as soon as the 1st month after one injection of 140 mg erenumab. Mean duration of headache episodes in EM decreased from 7.8 h at baseline to 6.3 h at 1 month, 6.3 h at 2 months, and 5.8 h at 3 months (all the timepoints *p* < 0.05). In CM, there was a numerical reduction in headache duration, but this reached the level of significance only during 2 months. Mean headache severity and the number of days with acute medication intake decreased significantly at all the time points, except for severity at 1 month in CM (Figure 4).

Three patients mentioned in their diary the occasional occurrence of “phantom” attacks without headache, but with the usual attack-related malaise, tiredness, “brain fog,” sensory hypersensitivity, and mild nausea.

**Figure 5:**
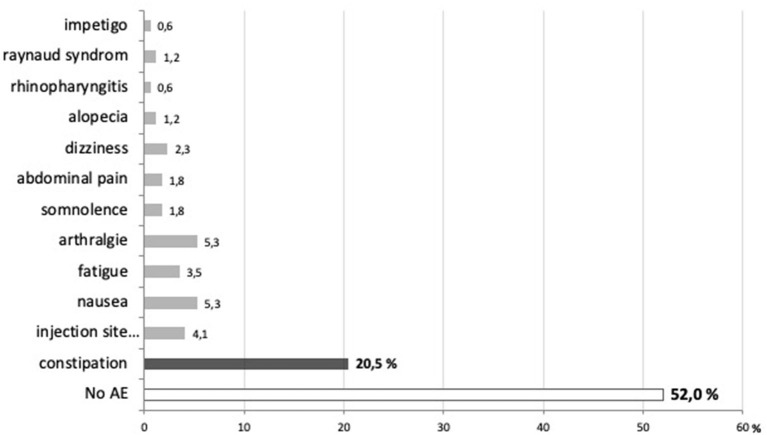
Proportion of patients reporting no (52%) or various adverse effects (48%) after 3 months of treatment with erenumab.

Tolerability of erenumab was good, as no serious adverse effect occurred. Forty eight percentage of patients reported non-serious, transient side effects, among which the most frequent were constipation (20.5%), nausea and arthralgia 24–48 h postinjection (5.3%), and short-lasting injection site reaction (4.1%) (Figure 5).

There was no new occurrence of arterial hypertension or aggravation of previous treated hypertension. We also found no significant weight change.

There were no drop-outs during the first treatment trimester and 75% of patients were satisfied with the treatment and would recommend it to others.

### Outcome After 12 Months

**Figure 6:**
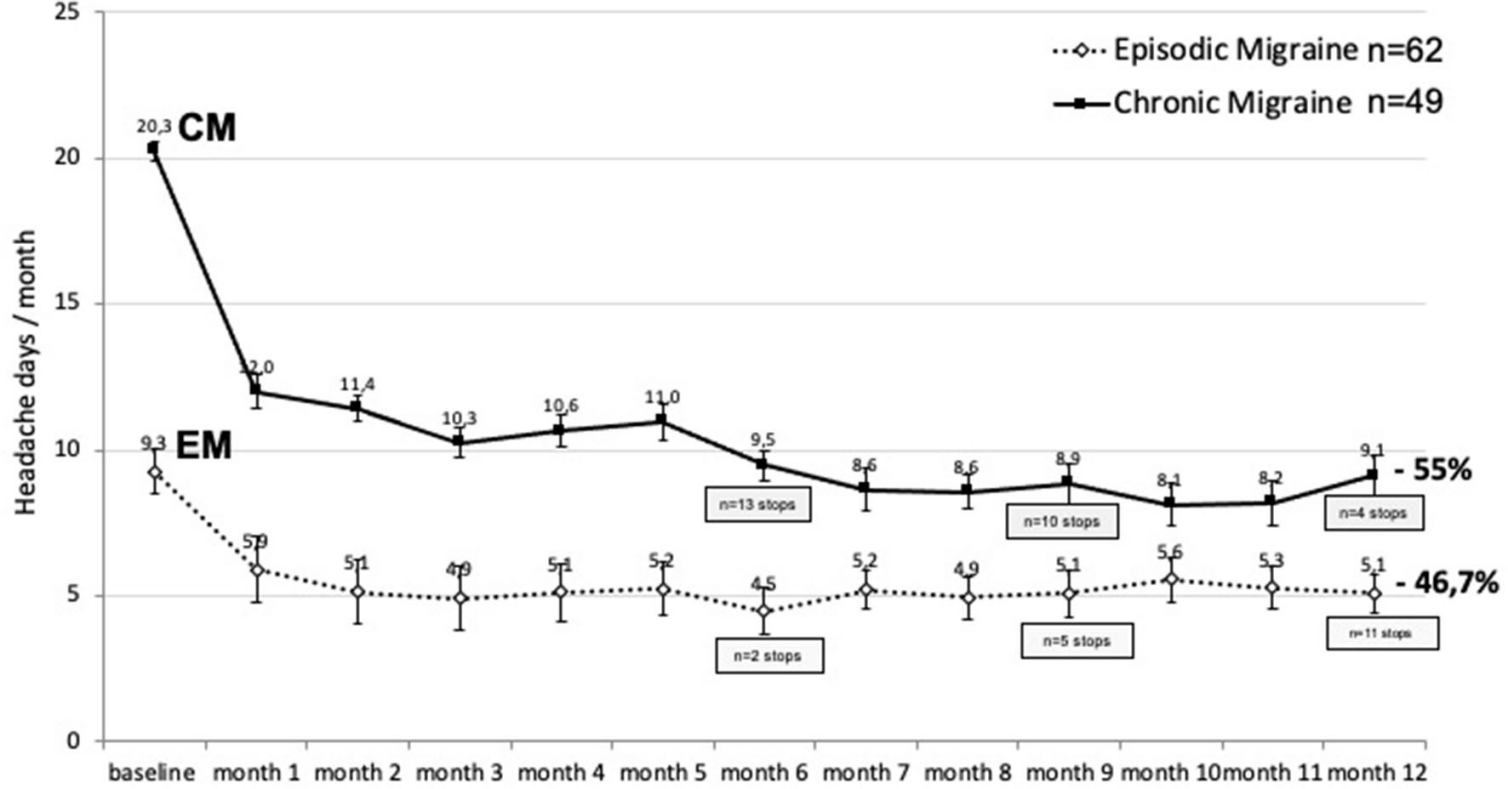
Monthly headache days during 12 months of erenumab treatment (mean ± SEM) and their percentage decrease during 12 months compared to the pretreatment month in EM (dashed line) and CM (continuous line). The number of patients who stopped treatment because of inefficacy or adverse effects is shown at 6, 9, and 12 months. *p* < 0.0001 vs. baseline at all the timepoints; *p* < 0.05 between EM and CM.

Figure 6 shows the evolution of mean monthly headache days in the 111 patients (EM, *n* = 62; CM, *n* = 49) who continued treatment up to 12 months (“per protocol”). The beneficial effect of erenumab persists over 1-year, but there is seemingly little supplementary benefit after 3 months. The slight MHD decrease after 5 months in CM is likely due to the fact that 13 patients with CM had abandoned treatment at the 6 month visit because of lack of efficacy (*n* = 9) or adverse effects (*n* = 4). This also contributes in CM to the greater MHD reduction during 12 months (55%) than during 3 months (35.5%) (see Figure 1).

In Table 3, the 50% responder rates at each timepoint have been calculated on an “intention-to-treat” basis, considering drop-outs for lack of efficacy or adverse effects as 0% response and discontinuation due to migraine remission as a ≥50% response.

**Table 3:** Retention of patients, 50% responder rates Intention-to-treat analysis (ITT), and reasons for discontinuation over 1-year of treatment with 140 mg erenumab monthly.

***TIMEPOINTS***	***Month 3***	***Month 6***	***Month 9***	***Month 12***
**EPISODIC MIGRAINE (ITT analysis)**				
Number of patients	80	78	73	62
**≥**50% responders (%)	44 (55%)	43 (54%)	41 (51%)	41 (51%)
Discontinuation due to lack of efficacy	–	2	4	4
Discontinuation due to adverse effects	–	–	1	1
Discontinuation due to migraine remission	–	–	–	6
**CHRONIC MIGRAINE (ITT analysis)**				
Number of patients	76	63	53	49
**≥**50% responders (%)	31 (41%)	34 (45%)	40 (53%)	31 (41%)
Discontinuation due to lack of efficacy	–	9	10	4
Discontinuation due to adverse effects	–	4	–	–
Discontinuation due to migraine remission	–	–	–	–

A total of 18 patients with EM stopped treatment between 3 and 12 months: 10 because they considered the treatment as inefficient and 2 because of severe constipation. Six patients felt that their migraine was not disabling anymore and withdrew from the program between 9 and 12 months. In the CM group, the reasons for discontinuation were lack of efficacy (*n* = 23, 30%) and, to a lesser degree, adverse effects (*n* = 4, 5%): two because of constipation, one because of skin lesions, which on biopsy were diagnosed as impetigo and probably not related to erenumab, and one because of nausea and arthralgias lasting for a week after the injection.

### Differences Between Clinical Phenotypes

We found no significant difference in the response to erenumab between patients with EM with low attacks frequency of 4–7 days/month (*n* = 22; 44% reduction of monthly headache days during 3 months) and those with high attack frequency of 8–14 days/month (*n* = 59; 45% reduction of monthly headache days during 3 months).

**Figure 7:**
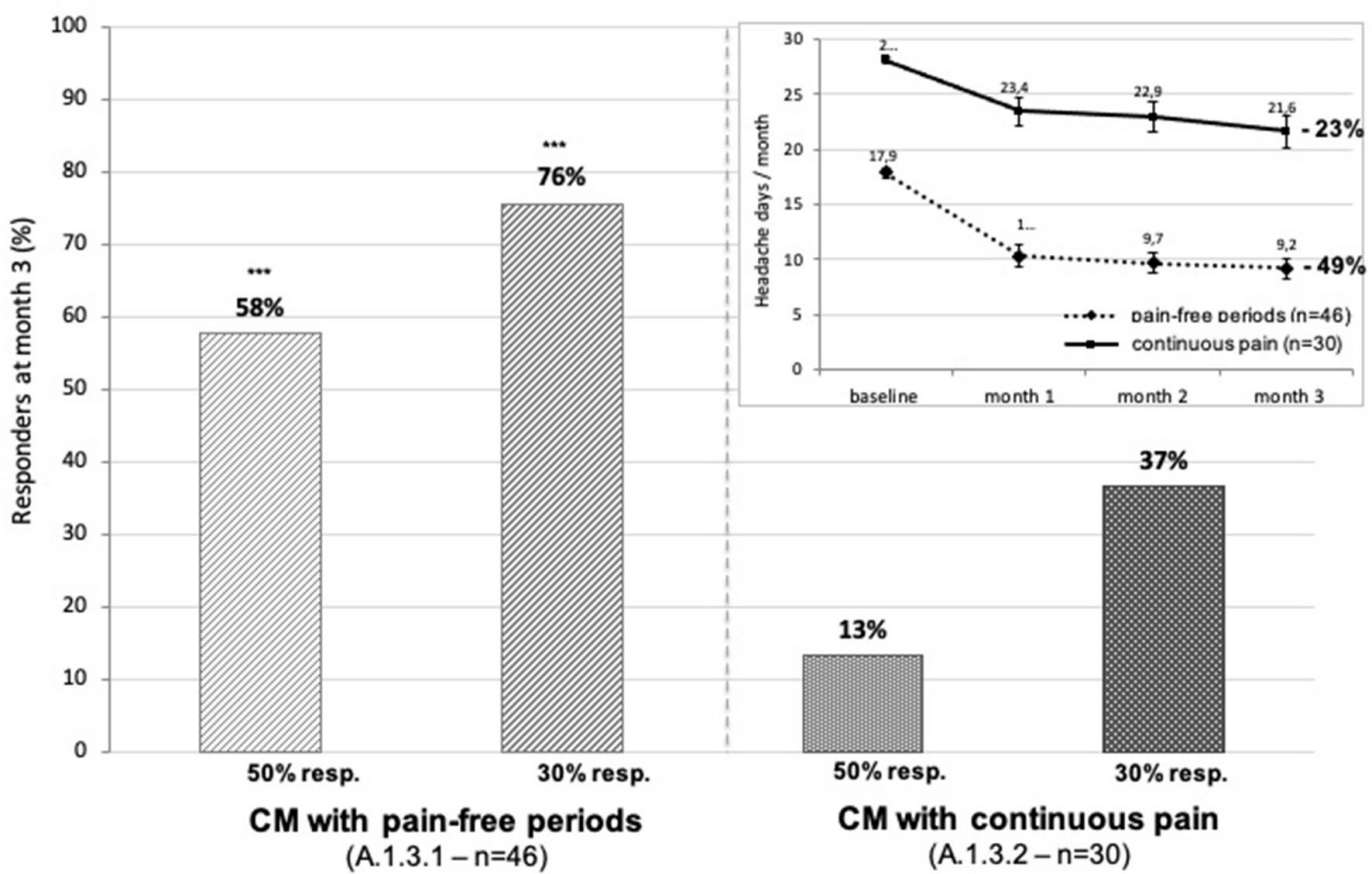
Proportion of patients with CM with 50 or 30% decrease in headache days during the 3rd month of erenumab treatment: on the left, patients with CM with pain-free periods (ICHD3 A1.3.1); on the right, patients with CM with continuous pain (ICHD3 A1.3.2). Insert: monthly change in headache days during the first 3 months of treatment and percentage decrease during 3 months (pain-free group: dashed line; continuous pain: continuous line). ***p < 0.001 chi-squared test “pain-free” vs. “continuous pain”.

By contrast, the most severely affected patients with CM with continuous pain (ICHD-3 A1.3.2) had no improvement after 3 months of treatment (13.3% of ≥50% responders), as opposed to patients with CM with pain-free periods (ICHD-3 A1.3.1) in whom the 50% responder rate was 58% (*p* < 0.001), i.e., comparable to that of patients with EM (55%). While the 30% responder rate was 76% in the latter, it was only 37% in the continuous pain group (*p* < 0.001) (Figure 7).

We found no significant difference in treatment response between patients with CM with (*n* = 50) and without acute medication overuse (*n* = 26). After 3 months, 56% of the former patients had withdrawn from medication overuse.

**Figure 8:**
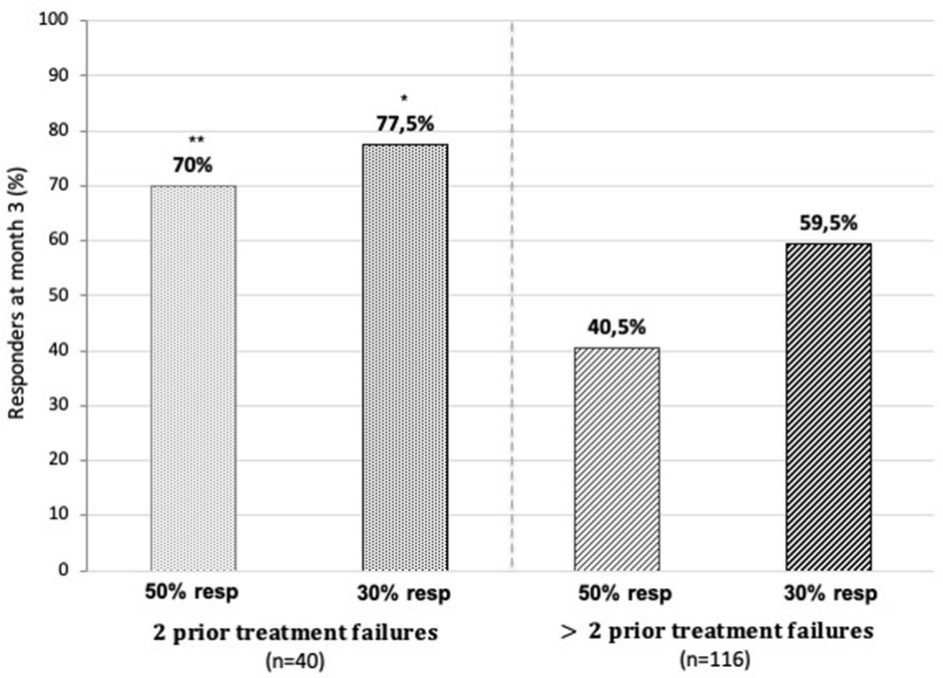
Fifty and 30% responder rates for monthly headache days at 3 months of treatment in patients with two prior treatment failures (on the left) and those with >two prior failures (on the right). *p < 0.05, **p < 0.01 chi-squared test “two prior failures” vs. “>two prior failures”.

When, at 3 months, patients with two prior prophylactic treatment failures (*n* = 40) were compared to those who had > two prior failures (*n* = 116), both the proportion of 50% (*p* < 0.01) and 30% responders (*p* < 0.05) were significantly lower in the latter subgroup (Figure 8).

**Figure 9:**
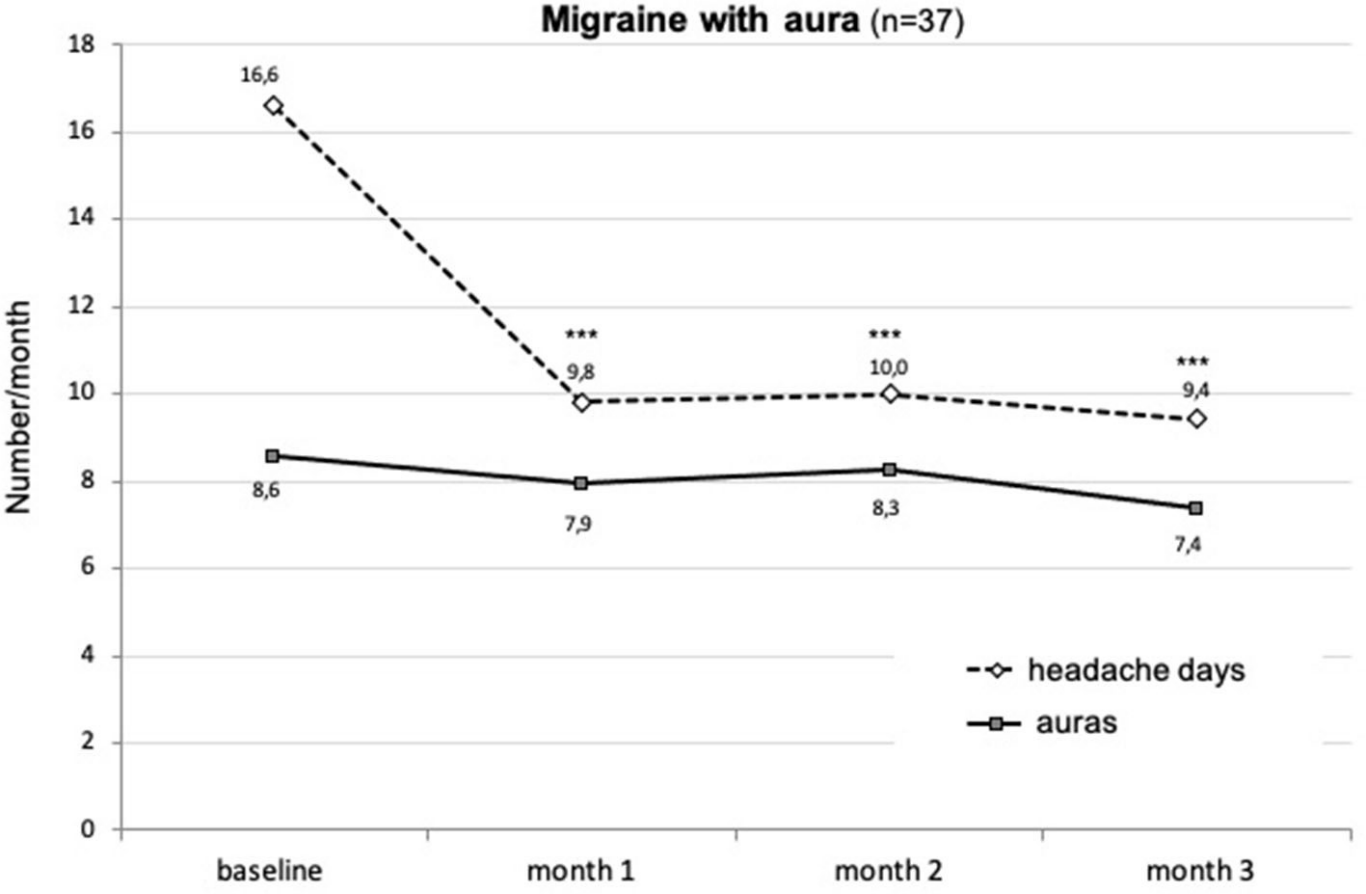
Change in monthly numbers of headache days (dashed line) and auras (continuous line) during the first 3 months of treatment with erenumab in patients having both migraine with and without aura attacks. ***p < 0.001 vs. baseline (Friedman's ANOVA, the Dunn's *post-hoc* test).

Finally, in patients also having migraine with aura (*n* = 37), treatment with erenumab significantly decreased MHD (*p* < 0.001), but had no effect on the occurrence of visual auras (Figure 9).

## Discussion

In this single center analysis of a compassionate use program that comprised several centers, erenumab at a monthly dose of 140 mg was highly effective as preventive therapy over the whole severity spectrum of migraine. The effect size, as judged by the 50% responder rates of 55% in EM and 43% in CM, was close to the respective rates of 50 (12) and 41% (14) found in the pivotal placebo RCTs that studied a monthly dose of 140 mg.

A number of “real-world” studies and registries of migraine treatment with erenumab in North American (18–25), European (26–33), or Australian patients (34) have been published recently and included mainly patients with CM (Supplementary Table 1). With one exception (21), all the studies from North America were retrospective (18–20, 22, 23, 26), while all studies but two (26, 30) studies from other geographic areas had a prospective design. The reported 50% responder rates for CM, although variable (range: 31.1–51.3%), are on average similar (45%) to that found in this study (44%). Only an Australian and two Italian studies stand out by higher rates of 58.8% (34), 55.5% (31), and 69.7% (28), respectively. The variability may be due to clinical differences between cohorts of patient, such as number of prior preventive treatment failures, and, thus, treatment refractoriness, duration of migraine chronicity and comorbidities, or to the use of an initial 70 or 140 mg monthly erenumab dose.

For patients with CM, a 30% reduction in monthly headache days is often considered to be clinically meaningful (35, 36). Nevertheless, 30% responder rates are rarely mentioned in “real-world” surveys. Raffaelli et al. (30) report such a responder rate of 51.1% in a cohort of 139 patients with CM. This rate is lower than the 60% rate found in this study, which might be explained by the fact that patients were more treatment resistant as they had failed on more than five prophylactic therapies including onabotulinumtoxin A.

Conversion from CM to EM is common with erenumab treatment. This occurred in 54.1% of patients with CM in our experience, which is comparable to the 52.7% reported in a German real-world study (26), but higher than the 39% found in a British study of highly refractory patients with CM (mean of 8.4 prior treatment failures) (27) and lower than the 68.1% reported by Ornello et al. (37) after 4–6 months of erenumab. Similarly, reduction of acute medication use below diagnostic thresholds for medication overuse headache (16) is frequent: 56% in this study and 46.9% in study by Scheffler et al. (26).

After 1-year of follow-up, we found drop-out rates due to lack of efficacy of 12.5% in EM and 30.2% in CM. In RCTs of erenumab, discontinuation of treatment during the double-blind phase is exceptional (12, 14). However, in the subsequent open-label extension follow-up, 34.5% of patients with EM discontinued monthly 70 mg erenumab after 1-year, 21% either because of lack of efficacy or because they requested so, which is not otherwise specified, but probably encompasses inefficacy (5). In real-world studies, drop-out rates for lack of efficacy range from 1.4–1.9 after 12 weeks (31, 32) to 40% after 6 months (19, 27). None of patients with CM withdrew because their migraine remitted, contrasting with 9.5% of patients with CM who did so in a Spanish registry (32).

Clinical predictors of treatment success have been explored in several studies. Responder rates with erenumab are, in general, lower in CM than in EM both in the RCTs and real-world studies (25) (see Table 1 and Supplementary Table 1). The RCT in CM did not include patients with continuous pain (A1.3.2) (14). Outcome in this CM subgroup varies between real-world studies. Robblee et al. (19) report in 58% (25/43) of patients with a 6 month follow-up and initially daily headaches at a significant reduction of 5.5 headache and migraine days. In other studies, the proportion of patients with daily headaches was reduced by half after 6 months of erenumab treatment (27, 34). These findings are at odds with ours showing very low 50 and 30% responder rates (13 and 37% respectively), which could be due in part to psychiatric comorbidities (38) that were not specifically assessed in our patients, but found to negatively influence outcome in another study (31).

Given that patients with HFEM are comparable to patients with CM with regard to disability (39), we expected a comparable treatment effect size in these two groups and, hence, a lower effect in HFEM as compared to low-frequency EM (LFEM). We could not confirm this in this study where there was no significant outcome difference between LFEM and HFEM, but such difference might have been missed because of the low number of patients (*n* = 22) in the former group. We are not aware of another study comparing treatment outcome with erenumab between LFEM and HFEM. However, Barbanti et al. (31) report a numerically higher 50% responder rate after erenumab in patients with HFEM (59.4%) as compared to patients with CM (55.5%), suggesting that outcome in HFEM might be close to that of LFEM.

The negative effect on outcome of the number of prior treatment failures shown in this study was also reported in some other real-world studies (24, 32). Although the LIBERTY RCT (15) was taken as evidence that erenumab is effective in patients with EM even after failure of two to four previous treatment failures, the reported 50% responder rate of 30% is clearly lower than in the pivotal RCTs for EM (50%) (12) or CM (41%) (14).

Some authors have found a less favorable outcome with erenumab in patients with CM with acute medication overuse (24, 32, 40). However, this was not the case in other studies (15, 26, 27) or in this study.

Other identified predictors of treatment success with erenumab are as follows: unilateral headache in EM (31), less severe disability (32, 33), absence of tension-type headache, good response to triptans (25), and higher baseline migraine frequency in CM (31, 33), although the opposite has also been reported (40).

As shown in Figure 9, there was no significant change in frequency (or in quality) of auras in patients (24%) who also had regular attacks with aura. This was expected because erenumab does not penetrate the blood–brain barrier (BBB) and, thus, not reach the visual cortex where cortical spreading depression (CSD), likely responsible for the aura, originates. Moreover, in rodents, another CGRP mAb, fremanezumab, did not prevent CSD induced by pinprick even after opening the BBB (41). Although in all the real-world studies, 30–40% of patients with auras were included and no attention has been paid to the effect of erenumab on the aura. In a retrospective study by Robblee et al. (19), the only exception is a comment by one patient who reported worsening of his auras. By the same token, in three of our migraine patients who never had an aura before being treated with erenumab, attacks with visual aura occurred from the first trimester of treatment onward. These attacks were rare and did not need any supplementary treatment. *De novo* visual aura was also reported in a patient treated with galcanezumab (42).

As in RCTs of erenumab, adverse effects were overall mild and transient in this compassionate use program. Globally, six patients (6.8%) dropped out because of an adverse event, the most frequent being constipation (four out of six patients). Surprisingly, constipation was reported in maximally 3.2% of patients in the erenumab RCTs. By contrast, it is the most prevalent side effect mentioned in most real-world studies where its incidence ranges from around 8 (31, 32) to more than 20% (18, 19, 27, 29, 33). The reason for the discrepancy between RCT and subsequent studies is not clear. Constipation may have been underreported in RCT. Constipation seems to occur less frequently with the CGRP-ligand mAbs (18). Erenumab-induced constipation could be related to the finding that the calcitonin gene *Calcrl*, which encodes the CGRP receptor component to which erenumab binds, is highly expressed in enteric neurons in mice (43).

A major limitation of this study is that we did not collect data on disability and quality of life in this study. Our major interest was indeed to compare efficacy and tolerability results in a real-world setting with those of pivotal RCT of erenumab where disability and quality of life scales were not primary outcome measures. There is little doubt, however, that erenumab markedly increased quality of life and decreased disability in most of our patients, given the marked changes in headache frequency and severity, the very low incidence of adverse effects, and the high rate of satisfaction with the treatment.

All data considered, erenumab, such as the other CGRP mAbs, stands out, when compared to the most effective classical prophylactic migraine treatments, such as topiramate, by an unprecedented efficacy over adverse effect profile rather than just by a superior efficacy (3, 11, 44, 45). This, however, has to be investigated by an appropriate comparative trial between the two classes of treatment. Such a trial comparing erenumab and topiramate has been completed and its results were presented at international congresses (Reuter et al. 7th EAN Congress June 2021, HER-MES trial) (46), but they have not yet been published in extenso. Interestingly, nonetheless, this comparative trial seemingly shows that erenumab is also more effective than topiramate for high-frequency episodic migraine, besides being better tolerated.

To conclude, in a compassionate use program of patients covering the severity spectrum of migraine from ≥4 migraine days/month to chronic migraine and having failed **≥** two classical prophylactic treatments, erenumab 140 mg monthly was highly effective over a 12 month follow-up. The 50% responder rate was around 50% in EM and 40% in CM on an intention-to-treat basis. The therapeutic effect was significantly lower in patients with CM with continuous pain and in patients with more than two prior treatment failures, but not in patients with CM with acute medication overuse or in EM with **≥**8 monthly attacks. Twenty one percentage of patients interrupted the program because of lack of efficacy, especially patients with CM (30%). Few patients (2.6%) withdrew because of non-tolerable adverse effects, primarily constipation. The results are comparable to those reported in real-world surveys or registries with some differences.

## Data Availability Statement

The raw data supporting the conclusions of this article will be made available by the authors, without undue reservation.

## Ethics Statement

Ethical review and approval was not required for the study on human participants in accordance with the local legislation and institutional requirements. The patients/participants provided their written informed consent to participate in this study.

## Author Contributions

JS has conceptualized the study, recruited and followed-up patients, contributed to the data analysis, finalized the figures, and written the manuscript. PG and RN have constructed the database, analyzed the data, performed the statistics, and prepared a first draft of figures. GT has recruited and followed-up patients, contributed to the literature search, and to the writing of the manuscript. AF has recruited and followed-up patients. MM has contributed to the data analysis and figure drafting. All the authors have read the manuscript and agreed on its content and form.

## Conflict of Interest

JS is an advisor for Novartis Benelux, Teva Pharma, Lundbeck and Man & Science; he has been a principal investigator in clinical trials sponsored by Novartis, Teva and Eli Lilly. GT has been an investigator in clinical trials sponsored by Novartis, Teva and Eli Lilly. RN and PG have been data managers in clinical trials sponsored by Novartis, Teva and Eli Lilly. The remaining authors declare that the research was conducted in the absence of any commercial or financial relationships that could be construed as a potential conflict of interest.

## Publisher's Note

All claims expressed in this article are solely those of the authors and do not necessarily represent those of their affiliated organizations, or those of the publisher, the editors and the reviewers. Any product that may be evaluated in this article, or claim that may be made by its manufacturer, is not guaranteed or endorsed by the publisher.

## Supplementary Material

Supplementary Table 1 A synopsis of “real-world” studies with erenumab.
