# Supplementary material for: Erenumab for Migraine Prevention in a 1-Year Compassionate Use Program: Efficacy, Tolerability, and Differences Between Clinical Phenotypes
Source: Front Neurol. 2021 Dec 10;12:805334. doi: 10.3389/fneur.2021.805334 (PMC8703164; doi:10.3389/fneur.2021.805334)
Supplement: Supplementary Table 1 — A synopsis of “real-world” studies with erenumab. [file Table_1.DOCX]

| **Study &**  **patients** | **Treatment modalities &**  **study type** | **50% responder rate** | **Adverse events** | **Drop-outs for inefficacy/AEs** |
| --- | --- | --- | --- | --- |
| **Cheng et al 2020**  CM (n=170)  >3 prior failures | 70mg/mth (11.8%)  140mg/mth (88.2%)  6 months  Prospective | 58.8% (mth 3)  46.5% (mth 6) | Constipation: 7/170 (4%) | 19.4%  (month 3) |
| **Ornello et al 2020**  EM (n=5)  CM (n=84)  ≥2 prior failures | 70mg/mth (56.6%)  140mg/mth (43.4%)  6 months  Prospective | 51.7% (mth 3)  56.1% (mth 6) | 22.5%  (constipation: 13.5%) | 14.6% |
| **Robblee et al 2020** EM (n = 5)  CM (n = 95) | 70mg/mth (65%)  140mg/mth (34.7%)  6 months  Retrospective | 54.8% (mth 6) | 71.3%  (constipation: 23.6%) | 27.7% |
| **Raffaelli et al 2020**  CM (n=139)  ≥5 prior failures (+BoNTA) | 70 mg/month  140 mg/month (7.3%)  3 months  Retrospective | 31.1% | 37.4%  (constipation: 18.7%) | 21.6% |
| **Scheffler et al 2020**  EM (n=26)  CM (n=74)  >5 prior failures | 70mg/mth  3 months  Retrospective | EM: 52.7%  CM: 41.9% | 42%  (constipation: 23.8%) | 1% |
| **Lambru et al 2020**  CM (n=162)  mean 8.4 prior failures | 70mg/mth  140mg/mth (53%)  Prospective | 35% (mth 3)  38% (mth 6) | 48%  (constipation: 20%) | 40% (mth 6) |
| **Russo et al 2020**  CM (n=70) | 70mg/mth  140mg/mth (30%)  6 months  Prospective | 53% (mth 3)  70% (mth 6) | 25.7%  (constipation: 23.9%) | 0% |
| **Kanaan et al 2020**  Migraine (not specified) (n=241) | 70 or 140mg/mth  Retrospective survey (May 2018-January 2019) | 62.7% continue erenumab, 73.9% found it helpful | 70%  (constipation: 43%) | 34.4% |
| **Alex et al 2021**  CM (n=77) | 140mg erenumab (n=46)  120mg galcanezumab  6 months (n=31)  Retrospective | 48.5% (mth 3)  35;4% (mth 6) | 69.6%  (constipation: 32.6% ere, 17.4% galcane) | 39.1% |
| **Barbanti et al 2021**  HFEM (n=103)  CM (n=269)  >3 prior failures | 70mg/mth  (140mg in 11%)  3 months  Prospective | HFEM: 59.4%  CM: 55.5% | 13.7%  (constipation: 8.8%) | 1.4% |
| **Talbot et al 2021**  CM (n=98)  Mean 5.5 prior failures (+BoNTA) | 70mg/mth  140mg/mth (57%)  11 months  Prospective | n.a.  mean MHD:  - 6.4 (mth 3)  - 6.8 (mth 6)  - 6.5 (mth 9) | n.a. | 24% (mth 11) |
| **Faust et al 2021**  CM (n=1034) | 70mg/mth  140mg/mth (72%)  9.3 months (mean)  Retrospective | 35% | 18.4% | 23.6% |
| **Belvis et al 2021**  n=210  CM (89.5%)  > 7 prior failures | 70mg/mth (67.6%)  140mg/mth (32.4%)  3 months  Prospective | 37.1% | 20%  (constipation: 8.1%) | 13.3% |
| **Torres-Ferrus et al 2021**  n=155  CM (87.1%)  > 3 prior failures | 140mg/mth (n=109)  (galcanezumab 120mg: n=46)  3 months  Prospective | 39.5% | 29%  (constipation: 20%) | 16.1% |
| **Baraldi et al 2021**  CM (n=111)  > 3 prior failures | 70 or 140mg/mth  12 months  Retrospective | 55.86% | 44.14%  (constipation 28.3%) | n.a. |
